# Plagiarism’s Poison: Avoiding Scientific Misconduct

**Published:** 2015-01-01

**Authors:** Kelley D. Mayden

**Affiliations:** Ms. Mayden is an oncology nurse practitioner at Southwest Virginia Cancer Center in Norton, Virginia, and an assistant professor of nursing at King University in Bristol, Tennesee.

The scientific body of literature is an integral part of oncology advanced practice that provides advanced practitioners (APs) with evidence for clinical decision-making and directly impacts patient quality of care and outcomes. Advanced practitioners not only utilize information from the literature but continue to make significant contributions to the existing body of knowledge. In both cases, it is crucial to reflect on the impact published information has on the scientific community at large and the end user of the information: the reader. Malice in publication, whether intentional or unintentional, fractures the scientific community by creating a sense of mistrust and may negatively impact patients’ views of trusted providers. One such malice is plagiarism.

Plagiarism is a form of ethical and scientific misconduct. It violates a code of honor between the author and the editor and the author and the reader. The consequences of plagiarism vary among editors and publications but can include article retraction, a publication ban on the offender, a report of the offense to an employer or a professional body, loss of research funding, loss of professional integrity, and possible loss of employment (Das & Panjabi, 2011).

Advanced practitioners in oncology must become proficient in ethical writing and avoiding plagiaristic practices. A number of tips and software tools are available to help authors avoid plagiaristic practices.

## SCOPE OF THE PROBLEM

As defined by the World Association of Medical Editors (2014), "Plagiarism is the use of others’ published and unpublished ideas or words (or other intellectual property) without attribution or permission, and presenting them as new and original rather than derived from an existing source. The intent and effect of plagiarism are to mislead the reader as to the contributions of the plagiarizer. This applies whether the ideas or words are taken from abstracts, research grant applications, Institutional Review Board applications, or unpublished or published manuscripts in any publication format (print or electronic)."

Once thought of as a rare phenomenon, plagiarism is increasingly detected during the peer-review and editorial process, resulting in an increasing number of retractions. A PubMed review as of May 3, 2012, listed 2,047 biomedical and life-science research articles as retracted; articles were retracted for misconduct in 67% of cases; error in 21% of cases; duplicate publication in 14% of cases; and plagiarism in 10% of cases (Fang, Steen, & Casadevall, 2012). A separate analysis of articles retracted between 2004 and 2013 revealed a steady increase in the number of articles retracted; 2,343 articles were retracted, with 584 retractions relating to plagiarism (Singh, et al., 2014). Article retraction occurs internationally, with high rates in Italy, Turkey, Iran, Tunisia, China, and the United States (Amos, 2013).

Plagiarism is likely a multifactorial issue involving the drive and desire for professional recognition, academic achievement, professional promotion, publication deadlines, and, in some cases, a misunderstanding of the principles of ethical writing and referencing. In a study of 1,000 nursing students (26% response rate), a cross-sectional survey demonstrated that 50% of the students did not make use of referencing resources, but this was rarely intentional and predominantly due to a deficit in skills (Greenwood, Walkem, & Shearer, 2014). An earlier study among 198 second-year Croatian medical students assigned to essay writing found that only 9% of students did not plagiarize; the average percentage of text plagiarized was 19%, which underscores the prevalence of the problem (Bilić-Zulle, Frković, Azman, & Petrovecki, 2005).

Different types of plagiarism exist (Table 1), and although all are a form of misconduct, a universal code of reprimand does not exist. For instance, self-plagiarism or text-recycling may not be considered as severe as duplicate publication or plagiarism of text, as the writer is not stealing from another person. Nonetheless, authors should be transparent when there is overlap by both citing reused text in a manuscript and alerting the editor upon article submission (Harriman & Patel, 2014). Often, originally submitted works are protected by copyright laws, and copyright infringement is possible when text is recycled from a previous work (iThenticate, 2011). Table 2 outlines tips for avoiding self-plagiarism.

**Table 1:**
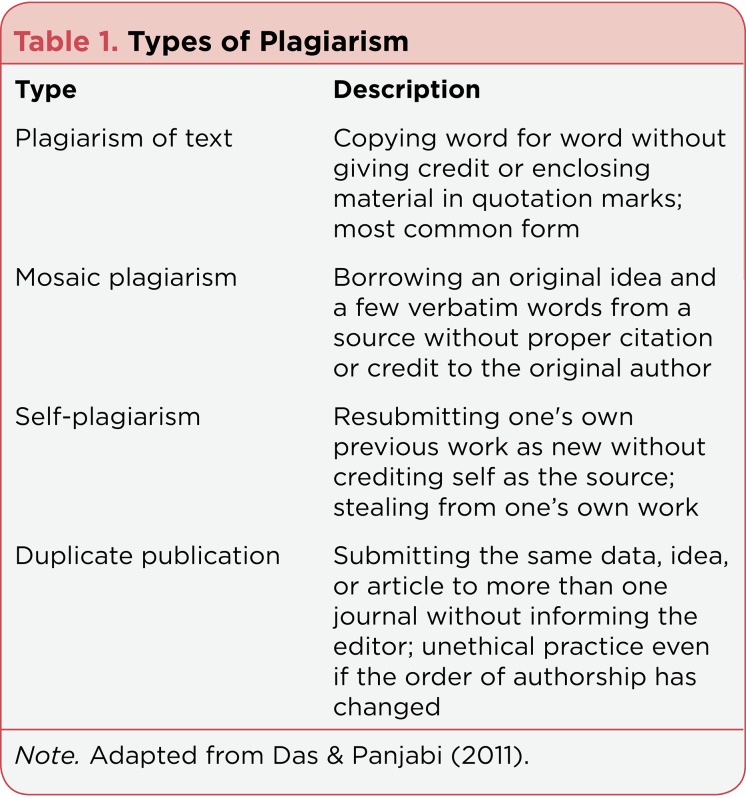
Types of Plagiarism

**Table 2:**
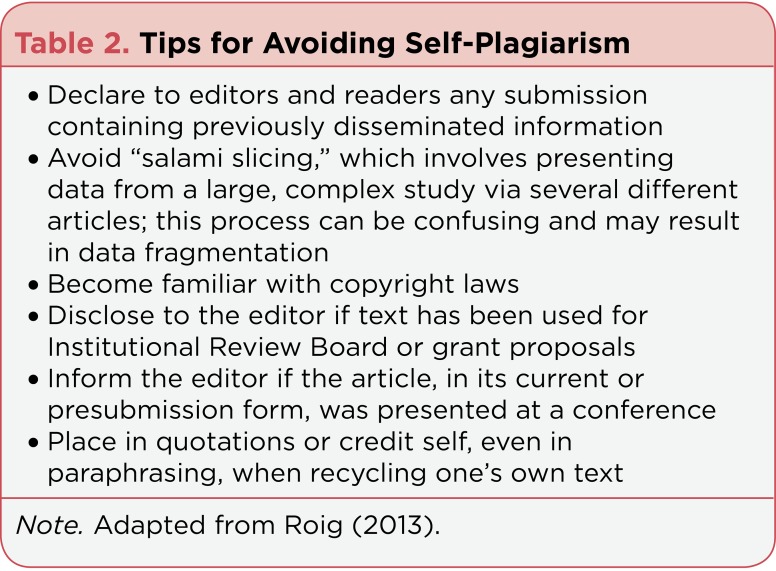
Tips for Avoiding Self-Plagiarism

## PREVENTING PLAGIARISM THROUGH INCREASED AWARENESS

An increasing awareness of the frequency and existence of plagiarism has resulted in efforts to reduce its occurrence at many levels. Academic institutions are incorporating writing ethics as part of the basic curriculum and have established centers for the surveillance, security, promotion, and development of quality research (Masic, 2014). Editors rigorously screen submissions for evidence of plagiarism and, in accordance with the Committee on Publication Ethics (2011), should employ all reasonable steps to ensure the quality of the material they publish. A system to detect plagiarism should be in place.

Peer review is another measure by which plagiarism may be detected or prevented. Peer reviewers have a responsibility to authors and editors to highlight suspected cases of plagiarism. Submission to a peer-reviewed journal offers an author a cross-check on content and references and can help derail allegations of misconduct and article retraction.

Ultimately, plagiarism prevention is an author’s responsibility. It begins with adherence to a high ethical standard by which the author will not allow himself or herself to misrepresent findings or to take credit for ideas generated by others. A number of organizations dedicated to ensuring the purity of scientific research provide information on the identification and prevention of plagiarism (Table 3). Table 4 outlines some guidelines and suggestions to help authors avoid plagiarism. The automatic scanning of manuscripts for plagiarism by the use of plagiarism detection software has become commonplace and is used by writers, editors, and researchers to improve the quality of scientific publishing (Kim, 2013). Multiple software tools are available; some tools can be accessed free of charge (Table 5).

**Table 3:**
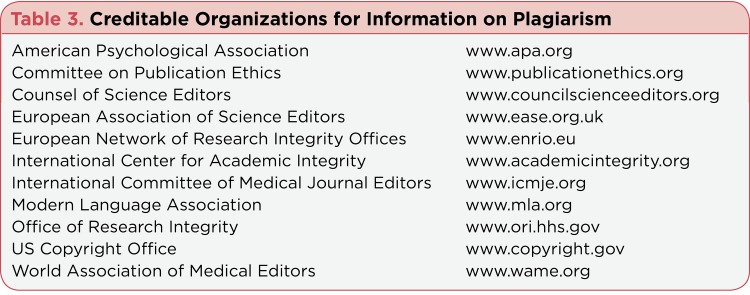
Creditable Organizations for Information on Plagiarism

**Table 4:**
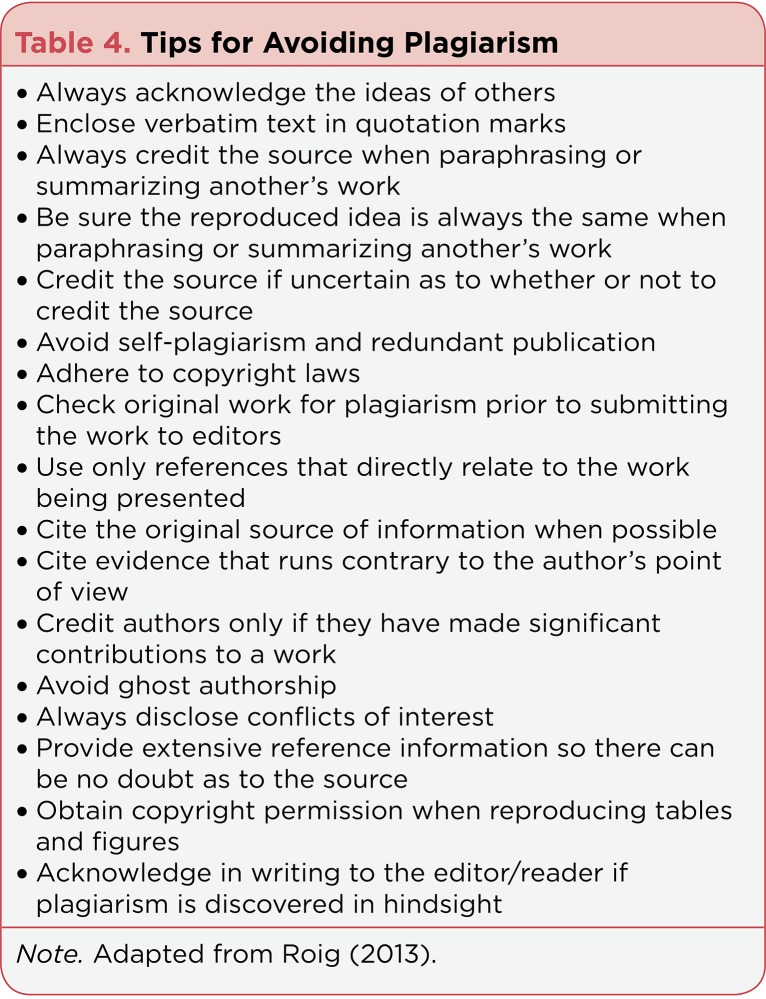
Tips for Avoiding Plagiarism

**Table 5:**
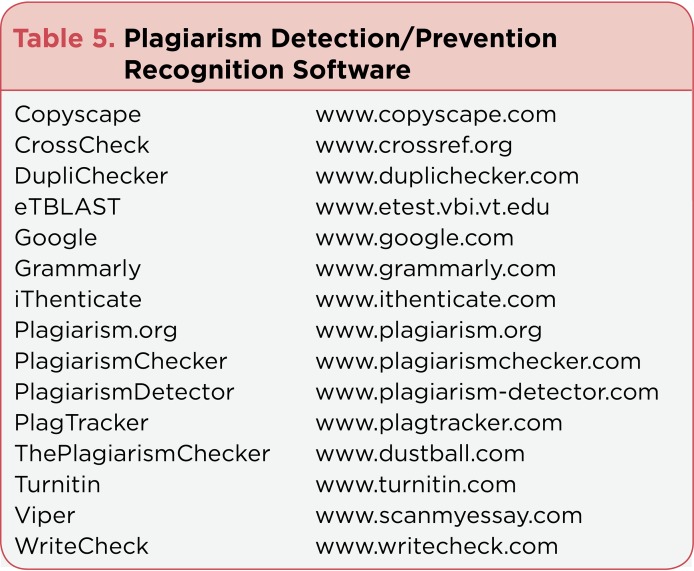
Plagiarism Detection/Prevention Recognition Software

Although there is limited evidence suggesting that plagiarism adversely affects patient safety, in the end, the practice of plagiarism is costly (Wardle, 2013). It increases editorial reviewing time frames, keeps critical research needed to care for patients out of the hands of practitioners, and wastes valuable time and money that could be spent on quality research (iThenticate, 2013).

## CONCLUSION

Plagiarism is a poison that ripples across the scientific body of knowledge, igniting mistrust among professional colleagues and information consumers. Knowing the potential plagiarism has to adversely affect patient outcomes and treatment paradigms by hindering the delivery of new information and wasting resources, oncology advanced practitioners must become proficient in ethical writing and avoiding plagiaristic practices. This can best be accomplished by presenting honest, reliable, and valid data and by always giving credit where credit is due.
